# Virtual resonance: analyzing IPA usage intensity under COVID-19's isolating canopy

**DOI:** 10.1038/s41598-024-64809-8

**Published:** 2024-07-01

**Authors:** Hyeon Jo, Eun-Mi Baek

**Affiliations:** 1 HJ Institute of Technology and Management, 71 Jungdong-ro 39, Bucheon-si, Gyeonggi-do 14721 Republic of Korea; 2 Department of Preventive Medicine, College of Medicine, The Catholic University of Korea, 222 Banpo-daero, Seocho-gu, Seoul, 06591 Republic of Korea

**Keywords:** Intelligent personal assistant, Artificial intelligence, Usage intensity, Parasocial interaction, COVID-19, Psychology, Mathematics and computing

## Abstract

The widespread adoption of smartphones coupled with advancements in artificial intelligence has significantly propelled the use of intelligent personal assistants (IPAs). These digital assistants have become indispensable for many users, particularly during the COVID-19 pandemic. Employing coviance-based structural equation modeling (CB-SEM) and analyzing data from 260 participants, this study explores the key factors influencing IPA usage intensity. Contrary to expectations, affective risk perception showed no significant impact on either IPA usage or parasocial interaction during the pandemic. In stark contrast, cabin fever syndrome significantly influenced both IPA usage and parasocial interaction, underscoring the role of environmental and psychological stressors in shaping technology use. Furthermore, loneliness was found to significantly enhance parasocial interaction with IPAs, though it did not affect usage intensity. The findings highlight a substantial connection between parasocial interaction and IPA usage intensity, suggesting that users who engage in human-like interactions with IPAs tend to use them more extensively. These insights not only deepen our understanding of how IPAs are utilized during health crises but also point to potential directions for developing IPAs that are more responsive to users' emotional and social needs.

## Introduction

The COVID-19 pandemic has profoundly altered daily routines, introducing widespread behavioral changes like mask-wearing, social distancing, telecommuting, and online schooling^1,2^. These adaptations have been pivotal in managing the spread of the virus but have also significantly impacted the psychological well-being of individuals worldwide. The pandemic has led to a notable decrease in physical interactions^3^, giving rise to various psychological challenges.

Among these challenges are increased feelings of fear and anxiety, stemming from regular updates about confirmed cases and fatalities^4^, as well as emotional responses to personal encounters with the virus. A prominent psychological impact has been cabin fever syndrome, characterized by frustration, restlessness, and irritability due to prolonged confinement^5–7^. This condition encompasses a range of emotional responses, including trauma, nihilism, and anxiety, as well as behavioral manifestations like severe procrastination and food cravings^8,9^. Moreover, the enforced social distancing has exacerbated feelings of loneliness and social isolation^10^, posing significant mental health challenges. To mitigate these psychological impacts, the role of social support has become paramount^11,12^. Social support, encompassing both verbal and non-verbal interactions, is crucial in reducing uncertainty, enhancing self-worth, and fostering a sense of belonging^13^. In this context, technology, particularly intelligent personal assistants (IPAs), has emerged as a vital tool in providing social support.

In the evolving landscape of Industry 4.0, marked by rapid technological advancements and digital transformation, IPAs have emerged as vital tools in providing not just operational assistance but also social support. IPAs, powered by artificial intelligence (AI), offer voice-based conversational services, assisting in a variety of tasks ranging from information provision to emotional support^14–17^. In the context of intelligent finance, similar to the complexities of the human nervous system, AI systems in treasury and finance management have evolved beyond mere task automation to encompass advanced data management and strategic decision-making processes^18^. This analogy extends to IPAs, which are becoming increasingly sophisticated, integrating and analyzing large datasets to provide more personalized and context-aware services. Additionally, as we delve into the phenomenon of Industry 4.0, it becomes imperative to understand the transformative role of technologies like IPAs in shaping a new societal and industrial paradigm. This includes considerations of automation, cost savings, and the broader impact on society and the global competitive landscape^19^. These developments underscore the importance of preparing society for the disruptive yet beneficial changes brought about by Industry 4.0 and the integral role of AI-driven tools like IPAs in this transition.

Studies have shown that users can form parasocial relationships with IPAs, engaging in interactions that mirror human relationships^20,21^. These advanced digital assistants, also referred to as AI assistants, virtual intelligent assistants, or voice personal assistants, are increasingly capable of recognizing and responding to users' emotional states^22,23^. This capability positions IPAs as not only functional tools but also as sources of emotional and social support, particularly beneficial in the current pandemic environment.

The ongoing COVID-19 pandemic has introduced a spectrum of psychological challenges, significantly impacting mental health and daily interactions. This study investigates how these challenges influence the utilization of IPAs. By exploring the specific roles and functionalities of IPAs, we aim to determine how these digital assistants can mitigate psychological difficulties and provide essential social support during these trying times. Our research contributes to a deeper understanding of the dynamic interaction between human psychological needs and technology, offering insights into how IPAs can be optimized to serve as supportive tools in managing the complex emotional landscape shaped by the pandemic.

The organization of this study is structured in the following manner: Section "Related research" elucidates the related research; Section "Theoretical Framework and hypothesis formulation" unveils the research model; Section "Methodology" elaborates on the methodology; Sect. 5 furnishes the research outcomes; Section "Discussion" encompasses a discussion concerning the results; while Section "Conclusion" provides insights for researchers and practitioners, along with the study's limitations.

Table 1 provides a list of acronyms used throughout the article, along with their full meanings to assist the reader in understanding the terminology employed.

**Table 1:** List of acronyms and their corresponding meanings.

Acronym	Meaning
AIC	Akaike information criterion
AGFI	Adjusted goodness of fit index
AI	Artificial intelligence
ARP	Affective risk perception
BIC	Bayesian information criterion
CB-SEM	Covariance-based structural equation modeling
CMB	Common method bias
CFS	Cabin fever syndrome
GFI	Goodness of fit index
IPA	Intelligent personal assistant
IUI	IPA usage intensity
LON	Loneliness
NFI	Normed fit index
PGFI	Parsimonious goodness of fit index
PSI	Parasocial interaction
RMSEA	Root mean square error of approximation
SRMR	Standardized root mean square residual
TLI	Tucker-Lewis index

## Related research

IPAs are AI-driven software designed to provide voice-based conversational services, transforming how users interact with technology in their daily lives^24,25^. These evolving capabilities of IPAs address a range of user needs, from performing tasks to offering social and emotional support^26,27^. This study examines the dynamics of user interaction with IPAs, especially in the context of the COVID-19 pandemic.

During the COVID-19 pandemic, the drastic reduction in outdoor activities and social interactions significantly influenced individuals' mental states^28–30^. This state of heightened anxiety and isolation can lead individuals to increasingly rely on IPAs as a source of comfort and information^31^. The usage of IPAs in such scenarios extends beyond conventional task-oriented interactions^32–34^. Individuals may turn to IPAs not only for information relevant to the pandemic, such as news updates or health guidelines, but also as a means of alleviating feelings of anxiety and isolation^35–37^. The formation of relationships with IPAs—where users start to perceive these digital assistants as companions or friends^38^—can emerge as a coping mechanism. This phenomenon is particularly pronounced in situations where human interaction is limited, as during the COVID-19 lockdowns. Thus, IPAs serve a dual role: as providers of critical information and as sources of emotional support, helping users navigate through the psychological impacts of prolonged isolation and uncertainty.

The COVID-19 pandemic has heightened users' affective risk perception, a psychological state reflecting their anxiety or concerns about exposure to risks^39,40^. Bae and Chang^41^ delved into the precursors of behavioral intent towards contactless tourism by broadening the scope of the theory of planned behavior, positioning affective and cognitive risk perceptions as primary influencers of attitude, subjective norms, and perceived behavioral control. They confirmed a notable association between affective risk perception, attitude, and behavioral intention. Similarly, Adiyoso and Wilopo^42^ utilized the theory of planned behavior to elucidate social distancing intentions, illustrating that risk perception amplifies the degrees of attitude, social norms, and perceived behavioral control. Researcher have crafted a research model to scrutinize the determinants affecting protective actions amidst the COVID-19 crisis, indicating that risk sentiments positively impact hygiene practices and social distancing adherence^43,44^. Combining previous studies, it's evident that affective risk perception of individuals impacts health-related behaviors. This altered state could lead to an increased reliance on IPAs for information and comfort.

The prolonged nature of the COVID-19 pandemic has precipitated continuous psychological discomfort among individuals, notably manifesting as cabin fever syndrome, a condition significantly spotlighted in recent studies^45–47^. Cabin fever syndrome, characterized by distress, irritability, and a claustrophobic sense of restlessness due to extended confinement in isolated spaces^47^, can intensify mental health disorders such as anxiety, depression, and paranoia^48^. Confronting these challenges, individuals, especially those grappling with severe cabin fever syndrome, are increasingly turning to IPAs^49,50^. IPAs serve as a readily accessible means to alleviate emotional distress^51–53^. Users often develop parasocial bonds with these digital assistants, viewing them as sources of companionship and support, which in turn amplifies their reliance and usage^54–57^. Furthermore, beyond cabin fever, the pandemic has significantly exacerbated feelings of loneliness, underscoring the vital role of social support in mitigating these effects^58^. In this era of isolation, IPAs extend beyond their traditional functional roles, emerging as pivotal tools for emotional support and fostering a sense of connectedness. This expanded role of IPAs, catering to emotional and social needs, mirrors the evolving dynamics of human-technology interaction, adapting to meet the unique psychological challenges presented by the pandemic's isolating circumstances.

Loneliness has been increasingly studied within the context of interactions between humans and digital agents, revealing its significant impact on the nature and depth of these relationships. Research suggests that loneliness can catalyze the development of parasocial interactions with digital agents, where users seek companionship from AI-driven technologies during periods of social isolation^21,59,60^. These interactions often mimic traditional social relationships, providing emotional support and reducing feelings of loneliness^61,62^. Moreover, the intensity of usage of digital agents like IPAs is positively associated with the level of loneliness experienced by users^63,64^. Research highlights how effectively designed IPAs can serve as social surrogates, enhancing user engagement and mitigating the adverse effects of loneliness^36,49^. These findings underscore the dual role of IPAs as both service providers and companions, which becomes particularly valuable in managing loneliness through enhanced parasocial interaction.

Research has focused on parasocial factors in studying IPA use. Han and Yang^20^ explored crucial elements impacting the enduring usage intent of IPA users, revealing that parasocial relationships hasten the engendering of satisfaction, which significantly propels continuance intention. A key focus of this study is the concept of parasocial interaction and relationships in the context of IPAs. While parasocial interaction refers to the superficial layers of user engagement with IPAs, encompassing aspects such as responsiveness and perceived reality^65^, parasocial relationships signify a more profound bond where the IPA is viewed as a favorite friend or even family^66^. The more IPA users use, the more likely they are to form a parasocial relationship. This study introduces parasocial interaction because it targets all users who have used IPA five times or more. Numerous findings affirm a tight linkage between satisfaction, sustained usage, and continuance intent^67,68^. Inferring from previous works^20,21^, it's deduced that parasocial interaction positively correlates with IPA usage vigor.

In conclusion, the extensive literature review conducted in this study provides a comprehensive understanding of the multifaceted role of IPAs in addressing the psychological impacts of the COVID-19 pandemic. The research question, focusing on how IPAs can mitigate the psychological challenges induced by the pandemic, is intrinsically linked to the themes emerging from the literature. Studies on affective risk perception, cabin fever syndrome, loneliness, and the formation of parasocial relationships with technology form the basis of understanding the potential of IPAs as both informational and emotional support systems. The literature underscores the increasing reliance on IPAs for coping with isolation, anxiety, and other mental health issues, highlighting their evolving role from mere task-oriented tools to empathetic, socially supportive agents. This linkage between the literature and the research question underpins the study's aim to explore and validate the expanded capabilities of IPAs as crucial components in managing the psychological well-being of individuals in pandemic-affected contexts. This interconnection sets the foundation for our investigation, guiding the analysis of IPA usage patterns and their impact on users' mental health during these unprecedented times.

## Theoretical framework and hypothesis formulation

The overarching theoretical framework for this study is grounded in the Media System Dependency Theory (MSDT)^69^, which posits that the more an individual depends on a media system for understanding, orientation, and play, the more important that media will be to the individual, especially in times of conflict or change. This dependency theory is applicable to the use of IPAs as individuals increasingly rely on these tools for information and emotional support during the COVID-19 pandemic. This research extends MSDT by integrating concepts from the Social Presence Theory (SPT)^70^ and the Computers Are Social Actors (CASA) framework^38^. SPT suggests that media differ in their ability to convey the presence of others and the immediacy of communication, which can affect the user's psychological state and interpersonal behaviors. CASA proposes that humans apply social rules and expectations to computer interactions as they would in human interactions. These theories support the study's focus on how affective responses, isolation, and the need for social interaction during the pandemic increase dependency on IPAs.

**Figure 1:**
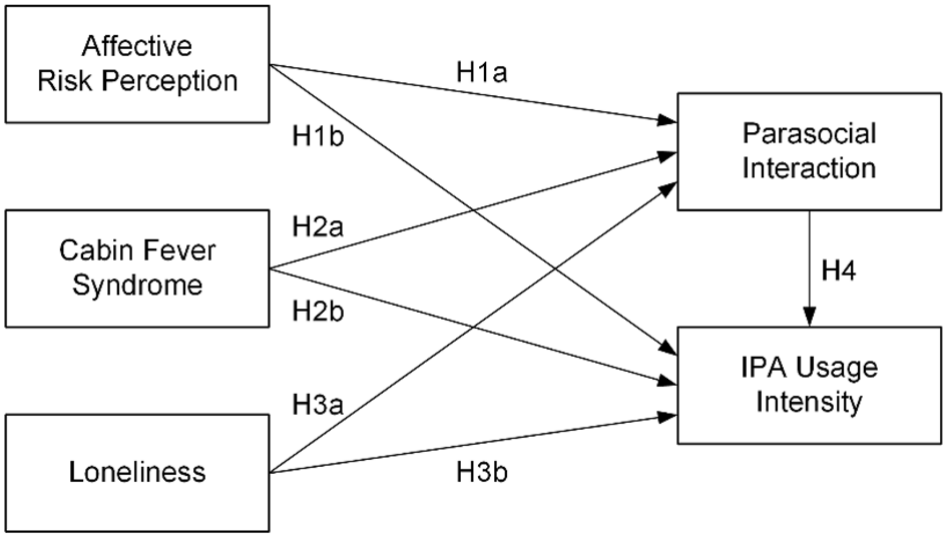
Research model.

Grounded in MSDT and integrated with SPT and the CASA framework, this research model outlines a comprehensive theoretical foundation for understanding user engagement with IPAs during the COVID-19 pandemic. This study hypothesizes that heightened affective risk perception during the pandemic, influenced by MSDT, leads to increased reliance on IPAs for coping and accessing information^71,72^. Similarly, cabin fever syndrome, characterized by restlessness and irritability due to prolonged confinement, is posited to increase dependence on IPAs for social presence and interaction, aligning with MSDT and SPT^73,74^. Additionally, increased loneliness, as explained by the CASA framework, is expected to lead to stronger parasocial interactions with IPAs, as users apply social behaviors to these interactions^75^. Finally, the combination of SPT and CASA suggests that parasocial interactions with IPAs fulfill social and emotional needs during isolation, thereby enhancing IPA usage intensity^76^. These hypotheses collectively elucidate how various psychological discomforts experienced during the pandemic amplify reliance on IPAs, impacting both the intensity of their use and the nature of user-IPA interactions. Figure 1 depicts the research model.

### Affective risk perception

Affective risk perception reflects an individual's anxiety or concerns about potential risks^77^. This perception often drives individuals to adopt protective behaviors like social distancing^42^. Furthermore, affective risk perception is positively linked to attitudes and behavioral intentions toward safer alternatives^41,43,78^. In the context of heightened risk perception, such as during the COVID-19 pandemic, individuals tend to reduce outdoor activities. This is where IPAs play a crucial role; they emerge as a reliable source of information, a comforting presence, or a distraction for individuals facing increased anxiety due to perceived risks. IPAs can provide up-to-date information, mitigate feelings of isolation through parasocial interaction, and offer solace in times of uncertainty. Therefore, this study proposes that affective risk perception not only increases parasocial interaction (H1a in Fig. 1) but also strengthens IPA usage intensity (H1b in Fig. 1), as IPAs become a go-to resource for those experiencing heightened risk awareness.

### Cabin fever syndrome

Cabin fever syndrome is characterized by irritability and distress experienced during prolonged isolation^6^. It often leads to a profound sense of restlessness and discomfort due to limited social interaction and physical confinement^79^. Individuals grappling with the symptoms of cabin fever syndrome may increasingly turn to IPAs as a source of comfort, distraction, or information. The IPAs, in this context, become a means to bridge the gap of social interaction, offering a semblance of companionship and an outlet to counteract the feelings of isolation^50,51,56^. This tendency to rely on IPAs for emotional relief and engagement in such situations is reflected in the formation of parasocial relationships with these virtual assistants (H2a in Fig. 1) and the increased usage intensity (H2b in Fig. 1). Consequently, this study suggests that the experience of cabin fever syndrome is directly linked to the development of stronger parasocial interactions with IPAs and the intensified use of them.

### Loneliness

Loneliness, defined as a subjective feeling of isolation, regardless of the actual level of social contact, can significantly influence an individual’s interaction with technology^80^. Studies in media psychology suggest that individuals experiencing loneliness may seek out social surrogates, such as television characters or virtual assistants, to alleviate their sense of isolation^21,55,81^. In the context of IPAs, this emotional state can lead to increased usage as individuals attempt to fulfill their social needs through these digital interactions^49,52^. Especially in the context of the COVID-19 pandemic, where social relationships are often curtailed, loneliness can intensify, driving individuals towards IPAs as a means of seeking companionship or interaction (H3a in Fig. 1). This leads to the formation of stronger parasocial relationships and an increase in IPA usage intensity, as they attempt to compensate for their lack of real-world social connections (H3b in Fig. 1).

### Parasocial interaction

Parasocial interaction is characterized by users feeling a sense of friendship or emotional connection with media figures, including those embodied by technology such as IPAs^60^. Research in human–computer interaction shows that when users perceive these digital agents as capable of social presence and responsiveness, they often engage more deeply and frequently with the technology^57,82,83^. This enhanced engagement due to parasocial interaction is hypothesized to increase the overall usage intensity of IPAs, as users find more value and satisfaction in these interactions, making them recurring aspects of their daily lives^84^. Therefore, the influence of parasocial interactions on increasing the usage intensity of IPAs is a focal point of investigation in this study (H4 in Fig. 1). Thus, this study posits that parasocial interaction significantly bolsters IPA usage intensity.

## Methodology

### Instrument formulation

The inquiries for our study were meticulously sourced from previously authenticated studies in related domains. In adapting these measurement markers for the IPA setting, careful modifications were made. For instance, while the original markers were primarily focused on general user interactions, we tailored them to more specifically highlight aspects unique to IPA interactions, such as voice recognition accuracy and response time. This refinement was essential to capture the nuances of user experience with IPAs. Each construct-related element was evaluated using a seven-point Likert scale, ensuring a comprehensive assessment. Furthermore, to validate these adaptations and their relevance to our research context, the questionnaire items underwent a thorough review by three esteemed scholars specializing in the management information system field.

Table 2 outlines the constructs and items used in the study, complete with their sources. The construct of affective risk perception includes three items expressing concerns about COVID-19, adapted from Bae and Chang^41^. For cabin fever syndrome, sourced from Chakraborty et al.^3^, there are three items that describe feelings of restlessness and social isolation during social distancing or lockdown. The loneliness construct, with three items sourced from Jang^21^, captures feelings of solitude and the absence of companionship. Parasocial interaction items, also from Jang^21^, depict the development of a personal connection with AI personal assistants. Finally, the IPA usage intensity construct includes three items that reflect increased usage of AI personal assistants amid social distancing or lockdown, sourced from Chakraborty et al.^3^. This table captures various dimensions of human-AI interaction and personal psychological states during the COVID-19 pandemic.

**Table 2:** Compilation of model constructs and items.

Construct	Item	Description	Source
Affective risk perception	ARP1	I am concerned about contracting COVID-19	Bae and Chang^41^
	ARP2	I am concerned for my family members contracting COVID-19	
	ARP3	I am concerned about the occurrence of COVID-19 in my area	
Cabin fever syndrome	CFS1	I feel restless remaining indoors during social distancing/lockdown	Chakraborty et al.^3^
	CFS2	I struggle to focus while staying at home during social distancing/lockdown	
	CFS3	I experience a sense of social isolation while staying at home during social distancing/lockdown	
Loneliness	LON1	I lack companionship	Jang^21^
	LON2	There is no one I can turn to	
	LON3	I feel alone	
Parasocial interaction	PIA1	I was able to form a personal connection with the AI personal assistant	Jang^21^
	PIA2	I frequently felt the AI personal assistant was reactive to me	
	PIA3	I frequently felt the AI personal assistant seemed genuine	
IPA usage intensity	IUI1	Amid social distancing/lockdown, I am utilizing the AI personal assistant more than usual	Chakraborty et al.^3^
	IUI2	Amid social distancing/lockdown, I am conversing with the AI personal assistant more frequently	
	IUI3	Amid social distancing/lockdown, I am employing functions via the AI personal assistant more than usual	

In our study, the measurement of variables (affective risk perception, cabin fever syndrome, loneliness, parasocial interaction, and IPA usage intensity) within the model and was based on self-reported data, where respondents themselves assessed and reported their feelings and experiences. This approach utilized standardized questionnaires, ensuring that each respondent's input reflected their personal perception and experience of these conditions.

### Participant demographics

Data collection was conducted through a well-established online survey firm based in South Korea. The inclusion criteria for participation in this study were specifically defined: participants had to have engaged with an IPA on at least five separate occasions prior to the study. Additionally, we established exclusion criteria to enhance the validity of our findings. Individuals who had never used an IPA or had used it fewer than five times were excluded, ensuring that respondents had a baseline familiarity and experience with the technology. The survey included questions to ascertain the frequency and context of respondents’ IPA usage, providing further insights into their interaction patterns with the technology. To ensure the integrity of our data, a rigorous process was employed to identify and eliminate insincere submissions. Submissions were considered insincere if they displayed patterns of uniform responses across diverse questions or exhibited extreme variability within variables that were expected to show consistency. This included instances where participants provided the same response to all items or where their answers varied excessively within a single scale, suggesting a lack of thoughtful engagement. After applying these criteria, 260 authentic responses were retained and subjected to further analysis. This step was crucial in maintaining the quality and reliability of our research findings.

The sample size for this study was determined using an a-priori sample size calculator for structural equation models^85^, taking into account the anticipated effect size, desired statistical power, number of latent and observed variables, and the probability level. Given an anticipated effect size of 0.1, a desired statistical power level of 0.8, 5 latent variables, 15 observed variables, and a probability level of 0.05, the minimum required sample size calculated was 200. Our study surpassed this requirement with a sample size of 260 participants, thus ensuring adequate power to detect statistically significant effects within the structural model. This sample size allows for robust statistical analyses and helps to ensure that the findings are reliable and generalizable within the context of the model's structure.

Table 3 discloses the demographic particulars of the participants. This study's demographic analysis presents a systematic overview of the respondents' characteristics. The average age of the respondents was 35.03 years (SD = 9.39), ranging from 14 to 59 years. Regarding gender distribution, it was fairly balanced with 131 males (50.4%) and 129 females (49.6%). In terms of IPA preference, 122 respondents (46.9%) used Bixby, followed by 108 (41.5%) using Siri, 26 (10.0%) using Google Assistant, 2 (0.8%) using Alexa, and 2 (0.8%) using other IPAs. The respondents' annual household income varied, with the largest group (33.1%, 86 respondents) earning between 30 and 50 million KRW. As for educational background, a significant majority held an undergraduate degree (181 respondents, 69.6%), followed by those who completed secondary school (44 respondents, 16.9%), with smaller percentages in other educational categories. This detailed demographic breakdown provides a comprehensive understanding of the study's sample.

**Table 3:** Sample characteristics.

Demographics	Item	Subjects (*N* = 260)	
		Frequency	Percentage (%)
Gender	Male	131	50.4
	Female	129	49.6
Age	10 s	10	3.8
	20 s	70	26.9
	30 s	96	36.9
	40 s	63	24.2
	50 s	21	8.1
IPA platform	Siri	108	41.5
	Bixby	122	46.9
	Alexa	2	0.8
	Google assistant	26	10.0
	Other	2	0.8
Annually household income (million KRW)	< 10	48	18.5
	10–30	51	19.6
	30–50	86	33.1
	50–70	53	20.4
	70–100	20	7.7
	> 100	2	0.8
Frequency	1 time per 3 months	24	9.2
	1 time per 2 months	9	3.5
	1 time per month	37	14.2
	2–5 times per month	58	22.3
	2–5 times per week	68	26.2
	1 time per day	23	8.8
	Several times per day	41	15.8
Education	In secondary school	9	3.5
	Completed secondary school	44	16.9
	Bachelor	181	69.6
	Master	22	8.5
	Doctor	4	1.5

### Control variables

In this study, demographic factors such as gender and age were meticulously controlled to prevent potential biases in the empirical analysis. Gender might shape the nature of parasocial interactions, with existing literature indicating gender-based differences in technology adoption and interaction patterns^86^. Age could be a critical factor influencing IPA usage, as younger individuals might demonstrate more tech-savviness and frequent interaction^87^. Additionally, the platform of IPA was a significant control variable, considering the diversity in voice recognition capabilities, response styles, and information processing algorithms among various IPAs^88^. For instance, popular IPAs like Siri, Bixby, and Google Assistant, incorporated in this study, differ in their technical architecture and user interface, potentially impacting user experiences and perceptions. These control variables are crucial in validating the study's outcomes, ensuring that any observed relationships are not merely reflections of demographic or technological variances but are intrinsic to the study’s primary variables.

### Ethical approval

This study was conducted in accordance with the guidelines of the Declaration of Helsinki. The research subject is unspecified, and the information collected through the research does not contain sensitive information in accordance with Article 23 of the Personal Information Protection Act of Korea and is exempted from IRB review.

### Informed consent

Informed consent was obtained from all individual participants included in the study.

## Research results

This study employed covariance-based structural equation modeling (CB-SEM) to rigorously test the hypothesized relationships within our research model. Chosen for its robustness in assessing latent constructs, CB-SEM is particularly adept at confirming theory-based expectations and causal pathways between variables, making it ideal for complex models like ours that aim to explore the psychological impacts of technology use^89^. The selection of CB-SEM was guided by its suitability for theory confirmation, especially in scenarios where precise estimation of relationships and overall model fit are critical^90^. This methodology allowed for a detailed assessment of the direct and indirect effects posited between factors within the research model, providing a comprehensive evaluation of the theoretical constructs proposed in the study.

### Common method bias (CMB)

CMB refers to the spurious variance shared among variables due to the measurement method rather than the constructs the measures represent^91^. It is essential to assess CMB to ensure the validity of research findings. In this study, CMB was examined through a single factor analysis, revealing that 48.090% of the variance could be attributed to a single factor. This figure suggests a potential presence of CMB, indicating the need for careful interpretation of the results^91^. Additionally, the analysis of the variance inflation factor (VIF) table (Table 4) unveiled relationships among variables such as affective risk perception, cabin fever syndrome, loneliness, parasocial interaction, gender, and age. The VIF values ranged from 1.049 to 2.049, indicating a modest degree of multicollinearity, which is within acceptable limits^92^. The findings from the single factor analysis, coupled with the VIF values, suggest that while there is some indication of CMB, it does not significantly compromise the study’s findings. Thus, the results are deemed robust and not substantially influenced by methodological biases.

**Table 4:** VIF value.

Construct	Parasocial interaction	IPA usage intensity
Affective risk perception	1.085	1.085
Cabin fever syndrome	1.833	2.049
Loneliness	1.742	1.833
Parasocial interaction		1.610
Gender	1.102	1.114
Age	1.095	1.172
IPA platform	1.049	1.081

### Measurement model

The assessment of the measurement model is a vital step in ensuring the validity and reliability of the constructs used in our study. This process involves evaluating item loadings, reliability, and both convergent and discriminant validity. The factor loadings, except for ARP2, ranged from 0.758 to 0.949, surpassing the recommended threshold of 0.7 and indicating satisfactory item reliability^89^. Although ARP2's loading was lower at 0.538, it was retained for its theoretical importance in understanding affective risk perception within the context of IPA usage. This item captures a nuanced aspect of the construct that other items may not fully represent, and excluding it could omit essential conceptual coverage. Reliability was further affirmed using Cronbach’s alpha, with values ranging from 0.748 to 0.959, exceeding the acceptable benchmark of 0.70^93^. For convergent validity, both Composite Reliability (CR) and Average Variance Extracted (AVE) were examined, with CR values above 0.70 and AVE scores exceeding 0.50, aligning with the criteria set by Fornell and Larcker^94^, thus corroborating convergent validity (Table 5).

**Table 5:** Descriptive statistics, reliability, and validity.

Construct	Items	Mean	St. dev	Factor loading	Cronbach's alpha	CR	AVE
Affective risk perception	ARP1	4.612	1.531	0.879	0.748	0.753	0.545
	ARP2	4.185	1.826	0.538			
	ARP3	5.035	1.418	0.758			
Cabin fever syndrome	CFS1	2.958	1.574	0.883	0.910	0.909	0.770
	CFS2	3.062	1.575	0.886			
	CFS3	3.185	1.630	0.863			
Lonliness	LON1	3.115	1.579	0.775	0.873	0.875	0.703
	LON2	2.631	1.567	0.911			
	LON3	2.981	1.735	0.824			
Parasocial interaction	PIA1	3.488	1.487	0.853	0.916	0.918	0.787
	PIA2	3.365	1.574	0.929			
	PIA3	3.012	1.535	0.878			
IPA usage intensity	IUI1	3.162	1.570	0.937	0.959	0.959	0.885
	IUI2	3.012	1.599	0.949			
	IUI3	3.096	1.567	0.936			
Gender	Gender	0.496	0.500	1.000	1.000	1.000	1.000
Age	Age	3.058	0.993	1.000	1.000	1.000	1.000
IPA platform	Platform	1.831	0.993	1.000	1.000	1.000	1.000

The assessment of model fit is essential to determine how well a proposed model represents the data collected. In this study, the covariance-based structural equation modeling (CB-SEM) technique was used to evaluate the fitness of the research model, which proposed the interplay of various psychological impacts on IPA usage intensity during the COVID-19 pandemic. The model produced a chi-square statistic of 188.975 with 123 degrees of freedom, which indicates that the model fit is reasonable^95^. A lower ratio of chi-square to degrees of freedom, 1.536 in this case, suggests a good fit, where values less than 2 or 3 are typically considered acceptable^90^. The RMSEA value was 0.045, with a 90% confidence interval ranging from 0.032 to 0.058. This indicates a close fit, as RMSEA values below 0.05 represent a good fit between the hypothesized model and the observed data^96^. The GFI was 0.927, and the AGFI was 0.898, both of which are above the commonly accepted threshold of 0.9, suggesting that the model adequately fits the observed covariance matrix^97^. The PGFI value was 0.667, which is acceptable, considering that values above 0.5 are generally deemed satisfactory^98^. The SRMR value was 0.051, further indicating a good fit, as values less than 0.08 are typically acceptable^99^. The NFI was 0.942, TLI was 0.974, and CFI was 0.979, all exceeding the recommended threshold of 0.9, which demonstrates a good fit to the data^100^. The AIC and BIC values were 284.975 and 455.888, respectively. Lower AIC and BIC values indicate a model with a better balance between goodness-of-fit and complexity^101,102^.

In summary, the model fit indices utilized in this study collectively suggest that the hypothesized model adequately captures the complexities of IPA usage in response to psychological discomfort during the COVID-19 pandemic. This supports the theoretical assertions posited in the study and underlines the importance of considering psychological factors when examining technology usage in crisis contexts.

### Structural model

**Figure 2:**
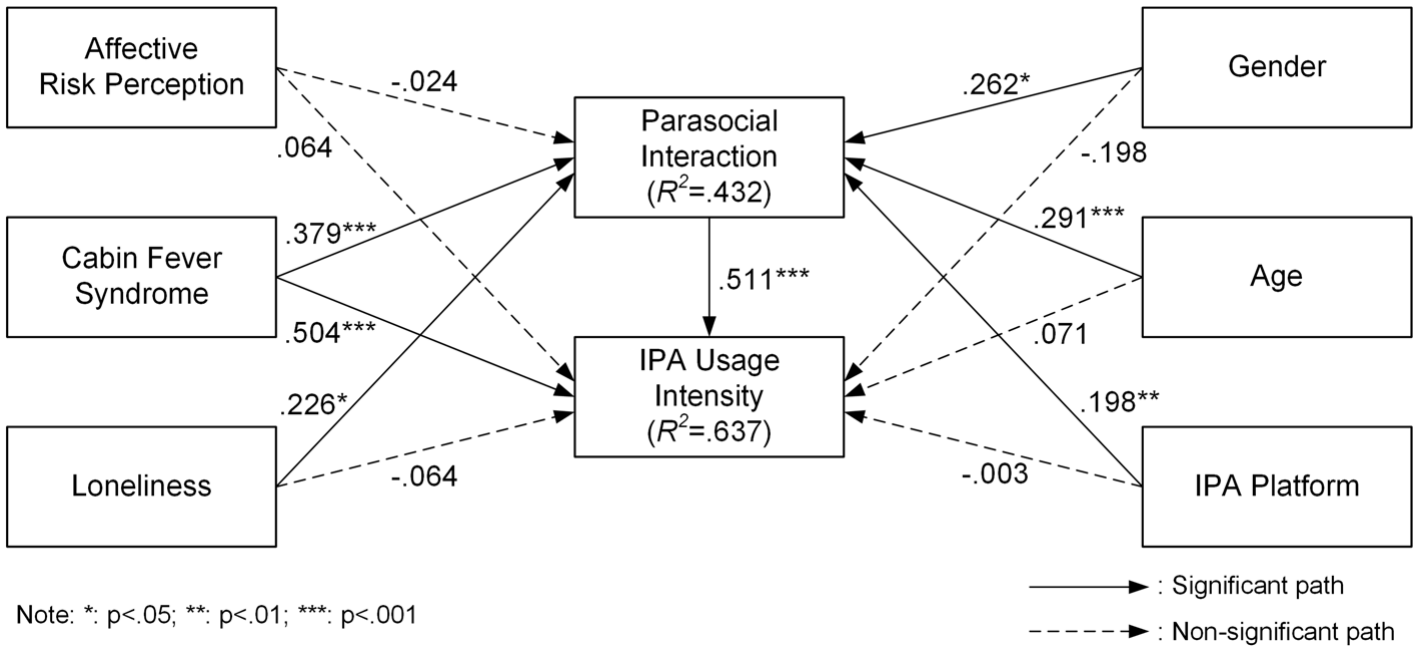
PLS algorithm results.

This investigation utilized a bootstrapping technique (subsample = 5000) to evaluate the stipulated hypotheses and path coefficients. Figure 2 illustrates the path coefficients along with the elucidated variances (*R*^*2*^) of the endogenous variables within the structural model. Out of the seven hypotheses postulated in the research model, four were substantiated. The outlined framework accounted for 43.2% of the variance in parasocial interaction and 63.7% of the variance in IPA usage intensity.

The results of the hypotheses testing using CB-SEM illustrate varied impacts across different constructs. For H1a and H1b, the influence of affective risk perception on parasocial interaction and IPA usage intensity was not supported, evidenced by coefficients of -0.024 (*p* = 0.388) and 0.064 (*p* = 0.234) respectively, indicating no significant impact. In contrast, H2a and H2b demonstrated that cabin fever syndrome significantly affects both parasocial interaction and IPA usage intensity, with coefficients of 0.379 (*p* < 0.001) and 0.504 (*p* < 0.001). These findings underscore cabin fever syndrome as a significant enhancer of both interaction and usage intensity among IPA users. Further analysis revealed that loneliness significantly impacts parasocial interaction (H3a), with a coefficient of 0.226 (*p* = 0.022), but does not significantly affect IPA usage intensity (H3b), where the coefficient was -0.064 (*p* = 0.282). H4 confirmed a strong positive effect of parasocial interaction on IPA usage intensity, with a significant coefficient of 0.511 (*p* < 0.001).

Regarding control variables, gender significantly influenced parasocial interaction (*β* = 0.262, *p* = 0.032) but not IPA usage intensity (*β* = -0.198, *p* = 0.068), while age affected parasocial interaction significantly (*β* = 0.291, *p* < 0.001) but not usage intensity (*β* = 0.071, *p* = 0.163). The platform of IPA also had a significant effect on parasocial interaction (*β* = 0.198, *p* = 0.002) but not on usage intensity (*β* = -0.003, *p* = 0.487). Table 6 summarizes the results.

**Table 6:** Results of hypothesis testing (CB-SEM).

H	Cause	Effect	*β*	*T*	*P*	Results
H1a	Affective risk perception	Parasocial interaction	− 0.024	0.285	0.388	Not supported
H1b	Affective risk perception	IPA usage intensity	0.064	0.724	0.234	Not supported
H2a	Cabin fever syndrome	Parasocial interaction	0.379	3.577	0.000	Supported
H2b	Cabin fever syndrome	IPA usage intensity	0.504	4.427	0.000	Supported
H3a	Loneliness	Parasocial interaction	0.226	2.015	0.022	Supported
H3b	Loneliness	IPA usage intensity	− 0.064	0.576	0.282	Not supported
H4	Parasocial interaction	IPA usage intensity	0.511	6.304	0.000	Supported
CV	Gender	Parasocial interaction	0.262	1.855	0.032	Significant
CV	Gender	IPA usage intensity	− 0.198	1.493	0.068	Not significant
CV	Age	Parasocial interaction	0.291	3.799	0.000	Significant
CV	Age	IPA usage intensity	0.071	0.981	0.163	Not significant
CV	IPA platform	Parasocial interaction	0.198	2.925	0.002	Significant
CV	IPA platform	IPA usage intensity	− 0.003	0.033	0.487	Not significant

*CV* control variable.

## Discussion

This study endeavored to scrutinize the principal elements influencing the intensity of IPA usage amidst the COVID-19 pandemic. The aim of this investigation has been realized by integrating the contexts of COVID-19 within the research framework.

The hypothesized influence of affective risk perception on parasocial interaction and IPA usage intensity (H1a and H1b) was not supported in this study, with *β* = -0.024, *p* = 0.388 for parasocial interaction, and *β* = 0.064, *p* = 0.234 for IPA usage intensity. These findings contradict previous research which suggested that increased risk perception during health crises could enhance engagement with technology for information and emotional support^39,40^. The minimal impact of affective risk perception observed in this study suggests that during the COVID-19 pandemic, other factors may play more substantial roles in influencing user behavior with IPAs. A possible interpretation might be that the immediate utility and emotional comfort provided by IPAs do not directly correlate with the heightened apprehensions about the pandemic, indicating that users may not turn to IPAs solely based on health risk concerns. This nuance in user behavior emphasizes the complex nature of technology adoption where direct causality may not be easily discernible.

Contrastingly, the results robustly supported the relationship between cabin fever syndrome and both parasocial interaction and IPA usage intensity (H2a and H2b), with significant coefficients (*β* = 0.379, *p* < 0.001 for parasocial interaction; *β* = 0.504, *p* < 0.001 for IPA usage intensity). These findings are in line with prior studies that recognized the role of prolonged confinement in escalating reliance on digital forms of interaction^36,103^. The substantial influence of cabin fever syndrome suggests that as individuals experience restlessness and isolation, they seek out IPAs not only as a source of information but also as social surrogates to alleviate feelings of confinement. This supports the notion that during extensive periods of isolation, such as lockdowns, users form stronger connections with digital assistants, fulfilling their need for interaction and reducing the psychological strain of isolation.

The analysis also revealed a significant but complex relationship concerning loneliness. While loneliness was found to positively affect parasocial interaction with IPAs (H3a, *β* = 0.226, *p* = 0.022), it did not significantly influence IPA usage intensity (H3b, *β* = − 0.064, *p* = 0.282). This partial support aligns with findings from previous works^60,104^, where loneliness was seen to increase the need for interaction, potentially enhancing parasocial relationships with non-human agents. However, the negative coefficient in H3b, although not significant, might suggest that while lonely individuals engage more with IPAs on a relational level, this does not necessarily translate into increased overall usage. This could indicate that the interactions may be more about quality and emotional depth rather than frequency or duration, pointing towards a nuanced understanding of how emotional states like loneliness interact with technology usage. These differentiated impacts of psychological factors on IPA interactions underscore the complexity of user engagement with technology, particularly in contexts altered by global health crises. The findings invite further investigation into the specific conditions under which emotional and psychological discomforts influence technology adoption and usage patterns, potentially guiding more tailored IPA applications that address distinct user needs during crises.

The influence of parasocial interaction on IPA usage intensity (H4) was strongly supported in this study, with a significant coefficient (*β* = 0.511, *p* < 0.001). This result corroborates earlier research that suggests a robust link between the development of parasocial relationships and increased interaction with technology^104,105^. The substantial coefficient indicates that users who develop a sense of friendship or emotional connection with their IPAs tend to use these systems more extensively. This enhanced engagement is likely driven by the perceived social presence and responsiveness of IPAs, which can fulfill emotional and social needs, particularly in contexts where human interaction is limited. This finding highlights the critical role of parasocial dynamics in shaping how users interact with digital assistants. As users perceive these AI systems not just as tools but as relational partners, their engagement intensifies, underlining the importance of designing IPAs that can effectively mimic human-like interactions to boost user satisfaction and retention. This significant relationship underscores the potential of integrating more advanced conversational capabilities and empathetic responses into IPAs to further enhance user experience and engagement.

The study reveals notable demographic effects on IPA interactions, particularly concerning gender. Significant differences emerge in parasocial interactions between men and women, with a positive correlation (*β* = 0.262, *p* = 0.032) indicating that women may engage more deeply with IPAs on a social level. However, gender does not significantly impact IPA usage intensity (*β* = − 0.198, *p* = 0.068), suggesting that while gender influences the nature of interactions with IPAs, it does not determine how frequently they are used. Age also plays a crucial role, particularly among older individuals who exhibit higher levels of parasocial interaction (*β* = 0.291, *p* < 0.001). This demographic tends to seek more companionship through IPAs, although this does not translate into higher usage intensity (*β* = 0.071, *p* = 0.163), indicating a preference for quality over quantity in interactions. Additionally, the platform of IPA, such as Bixby versus Siri, significantly influences parasocial interactions (*β* = 0.198, *p* = 0.002) due to Bixby's superior recognition and engagement features^106^, although it does not affect usage intensity (*β* = -0.003, *p* = 0.487). These insights emphasize the importance of tailoring IPAs to meet the diverse emotional and social needs of users based on demographic variables.

## Conclusion

### Implications for researchers

This research enriches the theoretical landscape of user interaction with IPAs by intricately examining how various psychological states influence usage patterns during the COVID-19 pandemic. This study extends beyond the conventional focus of previous research on user satisfaction and task efficiency^20,107^ to explore deeper emotional and psychological underpinnings of technology usage.

Firstly, the findings challenge the prevailing assumptions from prior works that heightened affective risk perception automatically translates to increased technology use for coping and information seeking^39,40^. Unlike past studies which mainly emphasized a direct correlation, this research uncovered that the impact of affective risk perception on IPA interactions is not significant. This suggests that users may not necessarily turn to IPAs as a primary resource in managing their pandemic-related anxieties. Instead, this outcome prompts a reevaluation of how IPAs are perceived in terms of their utility in crisis contexts, pushing scholars to investigate other potential psychological or contextual factors that might influence this relationship. It invites further scholarly exploration into the specific attributes of IPAs that could make them more appealing or effective as emotional support tools during crises.

Secondly, the research substantiates the significant role of cabin fever syndrome in increasing both parasocial interaction and IPA usage intensity, which aligns with and expands upon findings from studies focused on digital interactions during confinement^36,103^. Previous research has not extensively explored how specific symptoms of prolonged isolation, such as cabin fever, directly relate to the use of IPAs. By demonstrating that cabin fever can lead to greater reliance on IPAs for social interaction, this study adds a critical dimension to the media system dependency theory by suggesting that IPAs can serve as significant social surrogates during extended periods of isolation. This has implications for scholars to further investigate how different types of digital tools can uniquely satisfy the social and emotional deficits caused by isolation.

Lastly, the nuanced analysis of loneliness presents a more complex picture than previously portrayed by existing studies. While previous research often posits that loneliness should uniformly increase both the quantity and quality of interactions with IPAs^60,104^, this study differentiates between the nature of interactions (parasocial) and the sheer frequency of use. This differentiation highlights that while lonely individuals seek deeper connections with IPAs, these interactions do not necessarily correspond with increased usage. This insight is crucial for scholars focusing on human–computer interaction, suggesting that emotional states can differently affect aspects of technology use, and that enhancing user experience may require focusing on the qualitative aspects of interactions rather than just increasing quantitative usage.

These contributions urge scholars to rethink existing models of media interaction during crises, considering not just the functional attributes of technology but also the complex psychological landscapes of users. Future research could benefit from incorporating these insights to design more empathetic and context-aware technological interactions, especially for populations experiencing heightened emotional and psychological challenges.

### Implications for practitioners

This study offers significant insights for developers, marketers, and service providers in the IPA sector, highlighting the increase in IPA usage during confinement due to enhanced parasocial interactions. Developers can capitalize on this trend by incorporating more human-like features and emotionally intelligent responses into IPAs, such as advanced natural language processing and sentiment analysis. These enhancements could transform IPAs from mere functional assistants to empathetic companions, providing emotional support during periods of isolation.

Moreover, the findings underline the importance of addressing the emotional and psychological needs of users in IPA design. This could involve designing more engaging user interfaces or programming IPAs to initiate check-in conversations, offer mood-boosting content, or suggest activities that mitigate feelings of loneliness or cabin fever. Moving beyond traditional technological focuses, this study integrates psychological factors such as affective risk perception, cabin fever syndrome, and loneliness, broadening the understanding of user experiences under extraordinary conditions like the COVID-19 pandemic. This holistic approach not only enriches the existing body of knowledge but also lays a groundwork for future research and development, enhancing the practical application and relevance of IPAs in diverse real-world scenarios.

Particularly insightful is the impact of cabin fever syndrome on both parasocial interaction and IPA usage intensity. This suggests that IPAs tailored to recognize and address symptoms of cabin fever syndrome could significantly increase user engagement and satisfaction. Developers might incorporate features enabling IPAs to provide conversational support and content tailored to alleviate the effects of prolonged isolation. Additionally, marketers can position IPAs as not only functional tools but also as empathetic companions crucial during isolation periods, potentially expanding their market reach by emphasizing personalized user experiences.

The study also reveals a crucial relationship between loneliness and increased parasocial interactions with IPAs, indicating a need for innovations in conversational content to alleviate loneliness. IPAs could be enhanced with algorithms that better understand and respond to user preferences and emotional states, improving interaction quality and user satisfaction. Ensuring user interactions with IPAs are consensual and respect privacy is essential, potentially involving mechanisms for users to permit data use for personalization. These advances could position IPAs as not just tools but as empathetic companions, meeting the growing demand for technologies that provide both functional and emotional support.

### Limitations and future research

This study, while providing valuable insights, also presents certain limitations and opportunities for future research. The focus on IPA usage during the COVID-19 pandemic offers a unique perspective but may limit the generalizability of the findings. The dynamics of human-IPA interaction observed here might differ in other societal or personal contexts. Furthermore, this research predominantly concentrated on individual user experiences, leaving the exploration of multi-user interfaces and interactions relatively unexamined. Future research could broaden its scope to include a variety of user groups, socio-economic backgrounds, and cultural contexts. Investigating how different demographics interact with evolving IPA technologies could reveal more universally applicable insights. Delving into qualitative research methods, such as user interviews or narrative analyses, would provide a deeper understanding of the nuances in human-IPA interactions. Additionally, the reliance on self-reported data in this study could be addressed in future research by incorporating more objective measures or combining self-reported data with behavioral or physiological indicators. This approach would enhance the validity and reliability of the findings. Moreover, expanding the research to include a wider spectrum of gender identities, particularly the LGBTQI + community, would offer a more comprehensive and inclusive perspective on IPA interactions.

## Data Availability

The datasets used and/or analyzed during the current study available from the corresponding author on reasonable request.
